# A possible case of offspring sex manipulation as result of a biased adult sex ratio

**DOI:** 10.1038/s41598-023-51131-y

**Published:** 2024-01-08

**Authors:** Ramiro S. Arrieta, Paula Cornejo, Bettina Mahler, Paulo E. Llambías

**Affiliations:** 1 Instituto Argentino de Investigaciones de Zonas Áridas, CONICET, Mendoza, Argentina; 2 Departamento de Ecología, Genética y Evolución, IEGEBA-CONICET, Facultad de Ciencias Exactas y Naturales, Universidad de Buenos Aires, Buenos Aires, Argentina

**Keywords:** Evolutionary ecology, Population dynamics

## Abstract

Although random meiosis should prevent the facultative adjustment of offspring sex ratio, theory predicts that females should produce more of the sex with the higher reproductive value. We reported a case of offspring sex ratio manipulation in grass wrens *Cistothorus platensis*. Males in better body condition would have higher reproductive value than females due to the potential for social polygyny and extra-pair fertilizations. On the other hand, local demography influences reproductive strategies in grass wrens as male abundance affects both social polygyny and extra-pair paternity frequencies. We evaluated whether females bias their brood sex ratio in response to adult sex ratio and nestling body condition (a proxy for female’s prospects of producing high-quality males). Females raised more male offspring when males were less abundant in the population (female-biased adult sex ratio). However, we found no relationship between nestling body condition and brood sex ratio, suggesting that females did not bias the brood sex ratio towards males when able to raise nestlings in better body condition. Taken together, our results provide the first suggestive evidence that female birds can manipulate their offspring sex ratio in response to the adult sex ratio.

## Introduction

Sex ratio theory intends to explain the observed variation in the proportion of male and female offspring in natural populations. How parents allocate their reproductive effort to the production of sons and daughters is a central question in evolutionary biology^1^. The study of variation in offspring sex ratio is fundamental for understanding the evolution of behavioral plasticity under physiological and genetic constraints^2^. However, explaining such variation across taxa remains a major challenge^3^.

In taxa with chromosomal sex determination (CSD), random meiosis should result in an equal probability of producing a son or a daughter, which leads to an offspring sex ratio close to parity. Although it is expected that CSD prevents facultative manipulation of the offspring sex ratio^4,5^, theory predicts that females should exert some control over the sex of their offspring to produce more of the sex with the higher reproductive value^6^. Indeed, several studies reported that female birds (i.e., the heterogametic sex) can adjust their offspring sex ratio to environmental and phenotypic conditions such as parental care, male attractiveness, maternal condition, territory quality, and food availability (reviewed in Refs.^3,7–12^). For instance, female tree swallows *Tachycineta bicolor* in better body condition tend to overproduce male offspring^13^. Female house wrens *Troglodytes aedon* paired with males with multiple nest sites in their territories bias their offspring sex ratio towards males^14^. Female blue tits *Parus caeruleus* produce more sons in their broods when paired with males with brighter ultraviolet coloration in their plumage^15,16^ (but see Ref.^17^). These studies reasoned that females would use environmental and/or phenotypic cues to manipulate the offspring sex ratio based on the reproductive value of both sons and daughters. Despite several studies reporting biases in the offspring sex ratio, others failed to find such evidence (e.g., Refs.^18,19, 20^). Furthermore, studies on different populations of the same species reported inconsistent results (e.g., blue tit Refs.^15–17,21^, collared flycatcher *Ficedula albicollis* Refs.^22,23^, barn swallow *Hirundo rustica* Refs.^18,24^), which makes the generality of facultative offspring sex manipulation in birds still an intensely debated issue^12^.

Individuals may also manipulate their offspring sex ratio in response to demographic variables that may affect their indirect fitness. The adult sex ratio (ASR, defined here as the proportion of adult males in the adult population) is a fundamental variable in demography, which influences mating competition, sexual roles, mating systems, and parental care^25^. Fisher^26^ reasoned that when males and females are equally costly to produce, frequency-dependent selection should favour a balanced offspring sex ratio. Under a biased ASR, this hypothesis predicts that females would increase their indirect fitness by producing more of the rarer sex in the adult population. To our knowledge, only three studies have evaluated whether ASR influences the offspring sex ratio in birds (great reed warbler *Acrocephalus arundinaceus*^27^, savannah sparrows *Passerculus sandwichensis*^28,29^, bobolinks *Dolichonyx oryzivorus*^29^). However, these studies found no support for such an effect.

In this study, we report a case of offspring sex ratio manipulation in a grassland passerine, the grass wren *Cistothorus platensis*. The grass wren is an excellent model to study offspring sex ratio manipulation as males have higher reproductive value than females due to the potential for social polygyny and extra-pair paternity^30,31^. Hence, it is expected that males would exhibit higher reproductive variance than females, and that males in better body condition would have higher reproductive value than females. On the other hand, demography seems to influence reproductive strategies as both social polygyny and extra-pair paternity frequencies are affected by male grass wren abundance^30,31^. We took advantage of a drastic variation in the ASR over three breeding seasons (2015–2017) to evaluate whether females biased their offspring sex ratio in response to adult sex ratio or nestling body condition (a proxy for female’s prospects of producing high-quality males). If females bias their offspring sex ratio to produce more of the rare sex, we expect a negative relation between ASR and brood sex ratio. However, if females bias their offspring sex ratio when able to raise high-quality males, we expect a higher proportion of males in broods (i.e., male-biased brood sex ratio) containing nestlings in better body condition.

## Results

We were able to sex 199 nestlings from 43 broods. Four nestlings belonging to four different broods could not be sexed as we failed to obtain PCR products from those individuals. In 2015, the ASR was biased towards females (binomial test, *p* = 0.04; Supplementary Table S1) and a significantly higher number of male offspring was produced in the broods (binomial test, *p* = 2.39e−3; Supplementary Table S1). Also, social polygyny was frequent; 16 out of 26 males (61%) bred with more than one female simultaneously. However, social polygyny was infrequent in 2016 and 2017 (range 0–29%).

**Figure 1:**
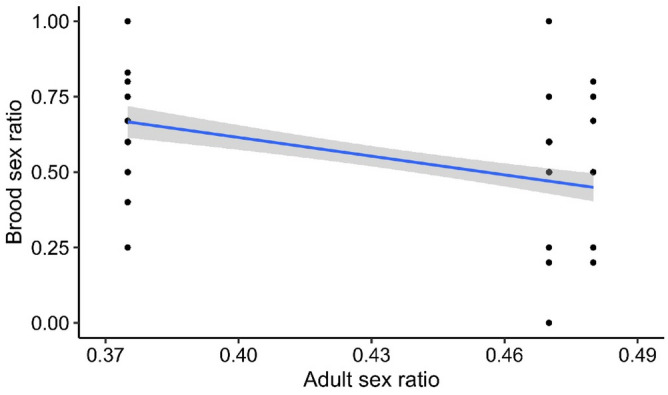
Relationship between brood sex ratio and adult sex ratio in south temperate grass wrens. The raw data (points) and model predictions with 95% confidence intervals (blue line and shaded area) are shown.

Nestling body condition was estimated from 195 nestlings. Brood sex ratio (defined in “Methods”) was not affected by nestling body condition (χ^2^ = 0.09, *p* = 0.75, Supplementary Table S2). However, we found a significant effect of the ASR on the brood sex ratio (χ^2^ = 6.14, *p* = 0.01; Supplementary Table S2; Fig. 1).

## Discussion

Our results strongly suggest that female grass wrens can manipulate their brood sex ratio. Since our analysis only included complete broods, departure from a binomial distribution was not caused by sex-differential mortality, suggesting that a physiological mechanism was involved^10,32^. Although our study only spanned over three breeding seasons, it is worth noticing that when breeding males were scarce, females consistently biased their brood sex ratio towards males (i.e., most sampled broods were male-biased). Accordantly, the brood sex ratio was negatively associated with the ASR.

Fisher’s^26^ hypothesis predicts that under a biased ASR, females would increase their indirect fitness by producing more of the sex that is rare in the adult population. The negative association between brood sex ratio and ASR suggests that female grass wrens may adjust their brood sex ratio to the rare sex. Since sex-differential mortality can be ruled out, the offspring sex ratio must have been biased within the female reproductive tract. Hormones circulating in the breeding female around the time of sex determination could influence the direction of sex allocation (reviewed in Refs.^10,32^). For instance, female spotless starlings *Sturnus unicolor* implanted with testosterone produce a higher proportion of sons in their broods than females receiving empty control implants^33^. Similarly, female zebra finches *Taeniopygia guttata* injected with testosterone during egg-laying tend to produce male-biased broods^34^. Studies integrating physiological, genetic, and ecological levels are needed to fully understand how these mechanisms work.

When the reproductive value of male and female offspring differs, theory predicts that parents should bias their offspring sex ratio towards the sex that provides the higher fitness gain^5,6^. Factors such as parental care, male attractiveness, maternal condition, territory quality, and food availability directly impact on female’s capability to produce nestlings in better body condition (reviewed in Refs.^3,12^). Several studies have documented that females can adaptively manipulate their offspring sex ratio in response to environmental and/or phenotypic conditions, improving the reproductive value of their offspring (reviewed in Refs.^10–12^). The influence of maternal condition on offspring sex ratio has been validated in many bird species, where mothers in good condition bias their investment towards producing sons (e.g., Ref.^10,35^). In grass wrens, females in good condition or/and in high quality territories would increase their fitness by producing high-quality sons. High-quality males should be able to monopolize more than one female to breed polygynously and/or to invest more time courting neighboring females to obtain extra-pair fertilizations. However, nestling body condition was not associated with offspring sex ratio, suggesting that females did not bias the brood sex ratio towards males when able to raise nestlings in better body condition.

To summarize, our study provides the first suggestive evidence that female birds may adjust their brood sex ratio in response to the ASR. While the consistent bias towards males across broods observed in 2015 points to an underlying factor that similarly affected most breeding wrens, the association between brood sex ratio and ASR suggests that a demographic variable may be involved. However, our results relied on a three years dataset and should be taken with caution. A bigger sample size spanning over several years will be needed to establish the validity of our results.

## Methods

### Study site

Fieldwork was conducted in the floodplain of the Uspallata Stream and Mendoza River (32° 38′ 10″ S, 69° 22′ 16″ W, 1800 m a.s.l.), Mendoza Province, Argentina. Our study site spanned approximately 120 ha and consisted of small swamps and pockets of riparian grasslands dominated by pampas grass *Cortaderia selloana*^36^. Seasonality is pronounced, with temperatures below freezing and occasional snowfall during the austral winter (mean temperature = 7 °C) and higher temperatures in the austral summer (mean temperature = 14 °C).

### Study species

The grass wren is a small (c. 9 g), insectivorous monomorphic passerine, broadly distributed throughout South America^37,38^. At our study site, grass wrens are year-round territorial and predominantly socially monogamous. However, social polygyny is frequent when breeding males are less abundant in the population^31,39^. Grass wrens present low to moderate rates of extra-pair paternity^30^. Both sexes collaborate in building the nest and in feeding the nestlings, but only females incubate the eggs and brood the young^31,40^. Clutch size ranges from 4 to 6 eggs and the incubation period is 14–18 days^40^. The nestlings remain in the nest for 12–19 days^39^.

### General field procedures

We carried out intensive fieldwork over three breeding seasons (from October to February 2015–2017). At the beginning of each season, we captured males with mist-nets by stimulating aggressive behavior with song play-back. Females were caught by herding them to the mist-net or by setting the mist-net close to the nest while they were feeding nestlings (8–12 days old). Adults were marked with a numbered aluminum ring and a unique combination of colored leg bands.

We monitored territories daily to determine social pairs and located nests by using parental behavioral cues (i.e., observing individuals with nesting material or with food to feed nestlings) and systematic searching. Thus, we were able to identify both social female and male from every nest. Nests were monitored until all nestlings fledged or the nesting attempt failed. We marked nestlings aged 7–8 days with numbered aluminum rings and collected blood samples. All blood samples were collected from the brachial vein and stored in lysis buffer until DNA extraction. When nestlings were 10 days old, we weighted them to the nearest 0.1 g on a 10 g Pesola Micro Spring Scale and measured their tarsus length to the nearest 0.1 mm with an SPI polymid dial caliper. We then estimated nestling body condition by using residuals from a regression of body mass on tarsus length.

Since some breeding pairs raised two broods during the same season, one brood of each breeding pair was chosen randomly for our sex ratio analysis. Thus, we avoided pseudoreplication in our dataset.

### Sex determination of nestlings

DNA was extracted following a standard protocol of dehydration and precipitation with ethanol and NaCl^41^. We determined nestling sex by using PCR amplification (2550F–2718R primers) of CHD1 genes located on the Z and W sex chromosomes^42^. PCR was carried out in a total volume of 10 µL with the following final reaction conditions: 10 mM TrisHCl pH 8.3, 1.5 mM MgCl2, 200 mM of each dNTP, 0.5 µM of each primer, 0.5 U of Taq polymerase, and 50–200 ng of genomic DNA. We performed PCR amplifications under the following thermal cycling conditions: initial denaturation (95 °C, 2 min), followed by 35 cycles of strand denaturation (94 °C, 30 s), primer annealing (45 °C, 30 s), and DNA extension (72 °C, 30 s). The program concluded with a final cycle of 72 °C for 2 min. PCR products were separated by electrophoresis for 30 min at 90 V in a 2.5% agarose gel stained with GelRed Nucleic Acid Stain (Biotium, Hayward, CA, USA) and visualized under UV light. Males were identified by the presence of a 700 bp band (Z-chromosome) and females by the presence of both a 700 bp band and a 500 bp band (W-chromosome). We validated the sexing procedure by using DNA from 6 adults of known sex (3 females and 3 males), and in all 6 cases our protocol correctly identified the sex of adults. Additionally, we confirmed that the sex assigned through this protocol as nestlings matched the sex assigned as breeding adults (based on behavioral and morphological traits) in 31 individuals.

### Adult and offspring sex ratios

Based on field observations of banded individuals and territorial boundaries, we calculated the ASR as the proportion of breeding adult males in the breeding adult population. We deemed as breeding adults all individuals (males and females) that occupied a territory. Field observations suggest that ‘floaters’ (i.e., non-territorial individuals) are uncommon in our study population as most males and females of known age (banded as nestlings) started breeding during their first year, and all adult birds that were captured in mist nets and banded were observed breeding or defending territories the year when they were captured.

We calculated the brood sex ratio as the proportion of male offspring in the brood. To correctly estimate the offspring sex ratio at the population level, it is necessary to prevent any potential biases resulting from sex-related mortality before sex determination^43^. Thus, only broods where mortality was zero (all eggs hatched and all nestlings survived until sampling) were included (43 out of 69). Additionally, a minimum brood size of three nestlings was chosen due to increased bias in broods containing fewer nestlings^44^.

### Statistical analysis

For each year, we determined whether both the offspring sex ratio at the population level and ASR deviated from parity by comparing the total number of male and female individuals using a binomial test.

We tested whether females adjust the brood sex ratio using a generalized linear mixed model with a logit-link function and binomial error distribution. The brood sex ratio was set as the response variable, correcting for brood size by binding the number of male and female nestlings per brood using the *cbind* command. Both nestling body condition and ASR were set as explanatory variables. Nest identity was included as a random factor to account for nonindependence of nestlings within broods. Neither father nor mother identities were set as random factors as only 13% of males and 7% of females were repeated among sampled broods. The lme4 package was used for this analysis^45^.

Analyses were performed in R^46^. The effects of nestling body condition and ASR on brood sex ratio were evaluated by comparing each model with a null model through likelihood ratio test against a chi-square distribution^47^.

### Ethical approval

Fieldwork permits (resolution 459 and 1564) were provided by the Secretaría de Ambiente y Desarrollo Sustentable, Dirección de Recursos Naturales Renovables, Mendoza, Argentina. All methods were carried out in accordance with relevant guidelines and regulations.

### Supplementary Information

Supplementary Information 1.

Supplementary Information 2.

## Data Availability

Authors can confirm that all relevant data are included in the paper and/or its supplementary information files.

## References

[1] West S (2009). Sex Allocation.

[2] Frank SA (1990). Sex allocation theory for birds and mammals. Annu. Rev. Ecol. Syst..

[3] West SA, Reece SE, Sheldon BC (2002). Sex ratios. Heredity.

[4] Maynard Smith J (1978). The Evolution of Sex.

[5] Charnov EL (1982). The Theory of Sex Allocation.

[6] Trivers RL, Willard DE (1973). Natural selection of parental ability to vary the sex ratio of offspring. Science.

[7] Sheldon BC (1998). Recent studies of avian sex ratios. Heredity.

[8] Hasselquist D, Kempenaers B (2002). Parental care and adaptive brood sex ratio manipulation in birds. Philos. Trans. R. Soc. Lond. B.

[9] Komdeur J, Pen I (2002). Adaptive sex allocation in birds: The complexities of linking theory and practice. Philos. Trans. R. Soc. Lond. B.

[10] Pike TW, Petrie M (2003). Potential mechanisms of avian sex manipulation. Biol. Rev..

[11] Booksmythe I, Mautz B, Davis J, Nakagawa S, Jennions MD (2017). Facultative adjustment of the offspring sex ratio and male attractiveness: A systematic review and meta-analysis. Biol. Rev..

[12] Navara KJ, Navara KJ (2018). Choosing Sexes: Mechanisms and Adaptive Patterns of Sex Allocation in Vertebrates.

[13] Whittingham LA, Dunn PO (2000). Offspring sex ratios in tree swallows: Females in better condition produce more sons. Mol. Ecol..

[14] Dubois NS, Dale KE, Getty T (2006). Surplus nest boxes and the potential for polygyny affect clutch size and offspring sex ratio in house wrens. Proc. R. Soc. B..

[15] Sheldon BC, Andersson S, Griffith SC, Örnborg J, Sendecka J (1999). Ultraviolet colour variation influences blue tit sex ratios. Nature.

[16] Griffith S, Ornborg J, Russell A, Andersson S, Sheldon B (2003). Correlations between ultraviolet coloration, overwinter survival and offspring sex ratio in the blue tit. J. Evol. Biol..

[17] Korsten P, Lessells CKM, Mateman AC, Van der Velde M, Komdeur J (2006). Primary sex ratio adjustment to experimentally reduced male UV attractiveness in blue tits. Behav. Ecol..

[18] Saino N, Ellegren H, Møller AP (1999). No evidence for adjustment of sex allocation in relation to paternal ornamentation and paternity in barn swallows. Mol. Ecol..

[19] Ewen JG, Cassey P, Møller AP (2004). Facultative primary sex ratio variation: A lack of evidence in birds?. Proc. Biol. Sci..

[20] Czyż B, Rowiński P, Wesołowski T (2012). No evidence for offspring sex ratio adjustment in Marsh Tits *Poecile palustris* breeding in a primeval forest. Acta Ornithol..

[21] Dreiss A, Richard M, Moyen F, White J, Møller AP, Danchin E (2006). Sex ratio and male sexual characters in a population of blue tits, *Parus caeruleus*. Behav. Ecol..

[22] Ellegren H, Gustafsson L, Sheldon BC (1996). Sex ratio adjustment in relation to paternal attractiveness in a wild bird population. Proc. Natl. Acad. Sci. USA.

[23] Rosivall B, Torok J, Hasselquist D, Bensch S (2004). Brood sex ratio adjustment in collared flycatchers (*Ficedula albicollis*): Results differ between populations. Behav. Ecol. Sociobiol..

[24] Romano A (2015). Sex allocation according to multiple sexually dimorphic traits of both parents in the barn swallow (*Hirundo rustica*). J. Evol. Biol..

[25] Székely T, Liker A, Freckleton RP, Fichtel C, Kappeler PM (2014). Sex-biased survival predicts adult sex ratio variation in wild birds. Proc. R Soc. B Biol. Sci..

[26] Fisher RA (1930). The Genetical Theory of Natural Selection.

[27] Bensch S, Westerdahl H, Hansson B, Hasselquist D (1999). Do females adjust the sex of their offspring in relation to the breeding sex ratio?. J. Evol. Biol..

[28] Wheelwright NT, Seabury RE (2003). Fifty: Fifty offspring sex ratios in Savannah Sparrows (*Passerculus sandwichensis*). Auk.

[29] Perlut NG, Travis SE, Dunbar CA, Strong AM, Wright DM (2014). Nestling sex ratios do not support long-term parity in two species with different life-history strategies. Auk.

[30] Arrieta RS, Campagna L, Mahler B, Lovette I, Llambías PE (2022). Local male breeding density affects extra-pair paternity in a south temperate population of grass wrens *Cistothorus platensis*. J. Avian Biol..

[31] Llambías PE (2020). Building multiple nests is associated with reduced breeding performance in a south temperate population of Grass Wrens *Cistothorus platensis platensis*. Ibis.

[32] Alonso Alvarez C (2006). Manipulation of primary sex-ratio: An updated review. Avian Poult. Biol. Rev..

[33] Veiga JP, Viñuela J, Cordero PJ, Aparicio JM, Polo V (2004). Experimentally increased testosterone affects social rank and primary sex ratio in the spotless starling. Horm. Behav..

[34] Rutkowska J, Cichoń M (2006). Maternal testosterone affects the primary sex ratio and offspring survival in zebra finches. Anim. Behav..

[35] Nager RG, Monaghan P, Griffiths R, Houston DC, Dawson R (1999). Experimental demonstration that offspring sex ratio varies with maternal condition. Proc. Natl. Acad. Sci. USA.

[36] Martínez Carretero E (2000). Vegetación de los Andes centrales de la Argentina. El valle de Uspallata Mendoza. Bol. Soc. Argent. Bot..

[37] Zarco A, Llambías PE, Medrano F, Barros R, Norambuena HV, Matus R, Schmitt F (2018). Atlas de las Aves Nidificantes de Chile.

[38] Remsen, J. V. *et al*. A classification of the bird species of South America. *American Ornithological Society* http://www.museum.lsu.edu/~Remsen/SACCBaseline.htm (2023).

[39] Llambías PE, Garrido PS, Jefferies MM, Fernández GJ (2018). Social mating system, male parental care contribution and life history traits of a southern Sedge Wren (*Cistothorus platensis platensis*) population: A comparison with northern Sedge Wrens (*Cistothorus platensis stellaris*). J. Ornithol..

[40] Llambías PE, Jefferies MM, Garrido PS, Fernández GJ, Reboreda J, Fiorini V, Tuero D (2019). Behavioral Ecology of Neotropical Birds.

[41] Miller SA, Dykes DD, Polesky HF (1988). A simple salting out procedure for extracting DNA from human nucleated cells. Nucleic Acids Res..

[42] Fridolfsson A, Ellegren H (1999). A simple and universal method for molecular sexing of non-ratite birds. J. Avian Biol..

[43] Fiala KL (1980). On estimating the primary sex ratio from incomplete data. Am. Nat..

[44] Bonderud ES, Flood NJ, Van Hamme JD, Boyda CA, Reudink MW (2016). Female mountain bluebirds (*Sialia currucoides*) paired to more colourful males produce male-biased broods. Behaviour.

[45] Bates D, Maechler M, Bolker B, Walker S (2015). Fitting linear mixed-effects models using lme4. J. Stat. Softw..

[46] R Core Team (The R Foundation, 2021).

[47] Crawley MJ (2007). The R Book.

