# Supplementary material for: A possible case of offspring sex manipulation as result of a biased adult sex ratio
Source: Sci Rep. 2024 Jan 8;14:819. doi: 10.1038/s41598-023-51131-y (PMC10774364; doi:10.1038/s41598-023-51131-y)
Supplement: Supplementary file 1 — Supplementary Information 1. [file 41598_2023_51131_MOESM1_ESM.docx]

**Supplementary information**

Manuscript title: “A possible case of offspring sex manipulation as result of a biased adult sex ratio”.

Author list: Ramiro S. Arrieta, Paula Cornejo, Bettina Mahler & Paulo E. Llambías.

Corresponding author: rarrieta@mendoza-conicet.gob.ar

**Table S1.** Sample sizes (number of individuals) and both adult and brood sex ratios of grass wrens for three breeding seasons (2015–2017).

|  | 2015 | 2016 | 2017 |
| --- | --- | --- | --- |
| Number of breeding males | 26 | 38 | 42 |
| Number of breeding females | 44 | 41 | 47 |
| *p*-value (binomial test) | **0.04** | 0.82 | 0.67 |
| Adult sex ratio | 0.37 | 0.48 | 0.47 |
| Number of broods | 18 | 11 | 14 |
| Number of nestlings | 85 | 48 | 66 |
| Number of male nestlings | 57 | 22 | 31 |
| Number of female nestlings | 28 | 26 | 35 |
| *p*-value (binomial test) | **2.39e-3** | 0.66 | 0.71 |
| Mean brood sex ratio*± SD | 0.66±0.22 | 0.45±0.22 | 0.46±0.29 |

*p*-value (<0.05) is indicated in bold.

*Calculated as the brood sex ratio averaged over all broods for that year.

**Table S2.** Result of a generalized linear mixed model analyzing the influence of the nestling body condition and adult sex ratio on the brood sex ratio over three breeding seasons in a population of grass wrens.

|  | Estimate ± SE | Z | *p*-value |
| --- | --- | --- | --- |
| Intercept | 4.77±1.77 | 2.70 | **-** |
| Nestling body condition | -0.06±0.19 | -0.31 | 0.75 |
| ASR | -10.34±4.04 | -2.56 | **0.01** |

*p*-value (<0.05) is indicated in bold.
